# Epigenetic Regulation in Mesenchymal Stem Cell Aging and Differentiation and Osteoporosis

**DOI:** 10.1155/2020/8836258

**Published:** 2020-09-10

**Authors:** Ruoxi Wang, Yu Wang, Lisha Zhu, Yan Liu, Weiran Li

**Affiliations:** Laboratory of Biomimetic Nanomaterials, Department of Orthodontics, Peking University School and Hospital of Stomatology, National Engineering Laboratory for Digital and Material Technology of Stomatology, Beijing Key Laboratory of Digital Stomatology, Beijing 100081, China

## Abstract

Mesenchymal stem cells (MSCs) are a reliable source for cell-based regenerative medicine owing to their multipotency and biological functions. However, aging-induced systemic homeostasis disorders *in vivo* and cell culture passaging *in vitro* induce a functional decline of MSCs, switching MSCs to a senescent status with impaired self-renewal capacity and biased differentiation tendency. MSC functional decline accounts for the pathogenesis of many diseases and, more importantly, limits the large-scale applications of MSCs in regenerative medicine. Growing evidence implies that epigenetic mechanisms are a critical regulator of the differentiation programs for cell fate and are subject to changes during aging. Thus, we here review epigenetic dysregulations that contribute to MSC aging and osteoporosis. Comprehending detailed epigenetic mechanisms could provide us with a novel horizon for dissecting MSC-related pathogenesis and further optimizing MSC-mediated regenerative therapies.

## 1. Introduction

### 1.1. Mesenchymal Stem Cells (MSCs)

MSCs are adult stem cells distributed in various mesenchymal tissues, which are derived from the mesoderm in the embryonic stage. MSCs exist in diverse tissues, such as bone marrow, umbilical cord blood, placenta, and adipose tissue [1]. Since MSCs are firstly isolated and defined from bone marrow, it has been traditionally accepted that bone marrow is the prevailing source of MSCs in humans [2, 3]. MSCs from different origins possess unique self-renewal capacities and can differentiate into multilineage cell types, including osteocytes, adipocytes, chondrocytes, and even endothelial cells or hepatocytes under certain given culture medium [4–6]. Apart from the aforementioned two basic characteristics, MSCs also exhibit various positive effects through paracrine action and immunomodulation during tissue repair, including regulating angiogenesis and osteoclastogenesis and guiding immune communication [7–9]. These properties signify that MSCs could perform extensive and active interactions with tissue-specific stem cell niches and represent an ideal and promising tool for tissue regeneration.

Although tentative therapeutic applications of MSCs have been carried out in the past years, disadvantages such as poor cell sources from diseased or aged hosts and *in vitro* passaging-induced senescent hypofunction both impair their therapeutic efficacy in tissue regeneration and hinder their large-scale clinical trials. MSC senescence manifests as division arrest, reflected by impaired proliferation and biased differentiation from osteoblasts towards adipocytes. Therein, biased differentiation can be induced by the imbalance between runt-related transcription factor 2 (*Runx2*) and peroxisome proliferator-activated receptor *γ* (*PPARγ*) pathway. These changes during senescence underlie bone mass loss and fat accumulation in aged or diseased skeletal tissues [10–13]. MSC aging is molecularly characterized by upregulated expression of senescence-associated genes such as *p53*, *p21*, *p16^INK4a^*, and *β-galactosidase* genes [14]. Notably, epigenetic regulation has emerged as a vital contributor to MSC aging and hypofunction, thus perturbing stem cell niche homeostasis and harming tissue health. Intriguingly, epigenetic alterations have also been demonstrated to modulate canonical senescence-associated genes directly or indirectly. Accordingly, therapeutic strategies based on epigenetic regulation may remedy tissue disorder in aging and diseases and further maximize the advantages of MSC-mediated tissue regeneration. In this review, we mainly focus on epigenetic marks and modifiers in regulating MSC aging *in vivo* or *in vitro*, in order to clarify the interactive link between epigenetic regulation and aging-related tissue diseases such as osteoporosis, and offer some clues for future utilization of epigenetics mediated tissue regeneration [15].

### 1.2. Epigenetic Regulation

**Figure 1:**
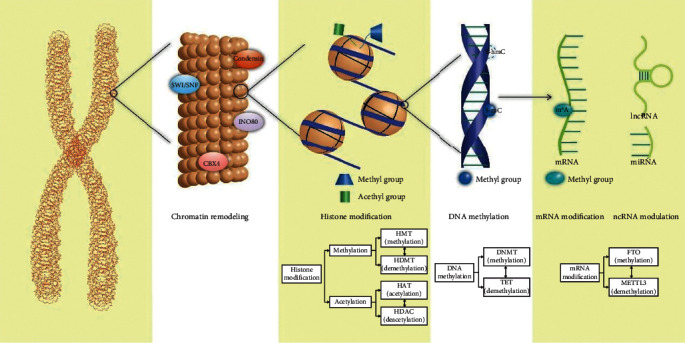
Factors in epigenetic regulation. Epigenetic regulation occurs in several steps of transcription (such as chromatin remodeling, DNA methylation, and histone modification) and posttranscription (such as mRNA processing and ncRNA regulation). Specific regulatory factors participate in each process.

Epigenetic regulation refers to altering phenotype through gene differential expression without changing DNA sequence and is a characteristic of heritability, reversibility, and no gene changes [11, 16]. Epigenetic alterations in cells happen in response to extrinsic environmental stimuli and cellular intrinsic inheritance to maintain cell and niche homeostasis. Accordingly, MSC aging or senescence *in vivo* or *in vitro* is also influenced by its own intrinsic dysregulation and microenvironmental factors from MSC niche, in the process of which typical epigenetic marks could be detected. In MSCs, epigenetic profile reflects dynamically transforming chromatin structure and corresponding transcriptional activity of genes; the major epigenetic mechanisms include DNA methylation, histone modifications, and chromatin remodeling [17]. In addition, posttranscriptional processing through mRNA and noncoding RNAs (ncRNAs) also takes part in epigenetic regulation of MSCs [18] (Figure 1). It has been widely documented that these epigenetic marks all have profound influences on MSC fate at multiple levels. Hence, further rationalizing and understanding the function mechanism of different epigenetic marks and modifiers occurring in MSC aging are of instructive importance to analyze the pathogenesis of aged and diseased tissue disorders and explore more effective therapeutic or regenerative strategies.

## 2. Epigenetic Regulation in MSC Aging and Osteoporosis

### 2.1. DNA Methylation in MSC Aging and Osteoporosis

DNA methylation refers to the covalent binding of methyl to the 5^th^ carbon of cytosine at CpG dinucleotide to form 5-methylcytosine (5-mC) under the catalysis of DNA methyltransferase (DNMT) [19]. This process can be reversed by demethylation relying on the catalysis of ten-eleven translocation protein (TET), which catalyzes the transformation of the 5^th^ carbon of cytosine into 5-hydroxymethylcytosine (5-hmC) [20]. In most cases, methylation of gene promoters or enhancers represents repressed expression (Table 1).

No matter *in vitro* or *in vivo*, alteration of DNA methylation profile gradually emerges as a close connection to MSC aging. Recently, researchers have successfully detected gene sites with methylation changes in the aging process by BeadChip microarray and found that alteration of methylation overlaps in aged MSCs *in vivo* and *in vitro* [21]. Afterward, in 2015, more than 10000 hypermethylation CpG sites and 40000 hypomethylation CpG sites were uncovered, many of which are associated with homeobox genes related to cell differentiation. For example, *Hox* and *Runx2*, as key transcription factors for osteogenesis, are hypermethylated in aged MSCs [21–23]. Moreover, in 2017, enhanced reduced representation bisulfite sequencing was used to depict a more precise DNA methylation profile, which finds that transcription factor binding sites (TFBS) for silent regulator 6 (*Sirt6*), E2F transcription factor 6 (*E2F6*), *JunB proto-oncogene*, and signal transducer and activator of transcription (*Stat5)* genes were hypermethylated along with the culture process, while TFBS for *Stat3* gene were hypomethylated. Besides, transcription factors influencing chromatin structure, such as *SMARCs* and *SIN3A*, are also differently methylated [24]. In general, the degree of methylation generally decreases in the process of aging [22]. On the other hand, these DNA methylation sites have also been discovered to be related to repressive and promotive histone modification, respectively. In MSCs, a large number of hypomethylated CpG sites are enriched in the region of active histone mark methylation of lysine 4 on histone H3 (H3K4me) indicating that H3K4 methylation is accompanied by DNA hypomethylation, and both of them are signs of increased transcription activity. By contrast, the hypermethylation CpG DNA region mainly overlaps with the repressive chromatin marks trimethylation of lysine 27 on histone H3 (H3K27me3), H3K9me3, and enhancer of zeste2 (EZH2), all of which play synergistic roles in inhibiting transcription activity [22, 25]. Particularly, H3K9me-promoted DNA hypermethylation of gene *p16^INK4a^* has also been proved to serve as a significant signature for epigenetic senescence in human MSCs (hMSCs) [26]. In MSCs isolated from aged individuals, 5-hmC also appears in specific CpG sites and, coincidentally, corresponds mostly to the hypomethylation region in the aged MSCs [27].

In the complicated regulatory network of DNA methylation, some regulatory factors and sites have been identified to have a direct relationship with MSC aging. For instance, 5-azacytidine (5-AzaC), as an inhibitor of DNMT1 and DNMT3b, exacerbates cell senescence by downregulating polycomb group proteins (PcGs) including B cell-specific Moloney murine leukemia virus integration site 1 (BMI1) and EZH2 through miRNAs. Since these PcGs are responsible for repressive mark H3K27me3 formation at the *p16* gene promoter, 5-AzaC finally facilitates *p16*'s activation in cell senescence. Moreover, inhibition of DNMT also directly demethylates cyclin-dependent kinase (CDK) inhibitor genes *p16* and *p21* [28]. However, 5-AzaC and another DNMT inhibitor RG108 were also reported to alleviate senescence by preventing reactive oxygen species (ROS) accumulation and maintaining telomerase reverse transcriptase (*Tert*) activity in aged human bone marrow MSCs (hBMSCs) [29, 30].

The methylation status of DNA is also responsible for differentiation potential and further influences the process of skeletal diseases. For example, in MSCs with high stemness, stemness-related transcription factors Octamer-binding transcription factor (OCT4) and NANOG protein both directly bind to the promoter region of DNMT1 and then transcriptionally activate it, followed by methylation and silencing of senescence-related genes [31]. On the contrary, when MSCs are treated with a DNMT1 inhibitor 5-AzaC, downregulation of DNMT1 is accompanied by hypomethylation of genomic DNA and increased expression of osteogenic genes such as *Runx2*, Osteocalcin (*Ocn*), distal-less homeobox 5, and Osterix (*Osx*), which is more visually presented by enhanced alkaline phosphatase (ALP) activity and mineralization [32]. Clinically, it has been reported that DNMT1 dysfunction also influences skeletal metabolic homeostasis. Aberrant CpG hypermethylation at gene ATP-binding cassette subfamily B member 1 (*ABCB1*, the encoding gene of P-glycoprotein) leads to osteonecrosis of the femoral head (ONFH) [33]. Besides, depletion of demethylase TET1 and TET2 causes osteopenia phenotype in mice by impeding demethylation of *P2rX7* promoter; *P2rX7* deficiency further leads to MSC incapability of exosome release, which results in intracellular accumulated miR-297 targeting Runx2 signaling pathway [34].

### 2.2. Histone Modification in MSC Aging and Osteoporosis

Histone modification is closely related to transcriptional activities of genes surrounding it. There are many different covalent modification types of the N-terminal amino acids of histone lysine, including acetylation, methylation, phosphorylation, ubiquitylation, and SUMO modification. Generally speaking, the first two are related to transcription activation, while the latter three often dominate transcription inhibition, with the exception of H3K4me3 [35]. In cases of aging and diseases related to MSCs, histone acetylation levels depending on the balance between histone deacetylases (HDACs) and histone acetyltransferases (HATs) and methylation levels relying on histone methyltransferases (HMTs) and histone demethylases (HDMTs) both make a critical difference [36, 37] (Table 2).

Histone modification regulates senescence by affecting the transcription activity of surrounding DNA related to cell cycle. For instance, the most direct regulators of repressive H3K27me3 can be divided into two categories: one is HMT such as G9a and PcGs including BMI1, EZH2, and SUZ12, and the other is HDMT such as lysine-specific demethylase 1 (LSD1) and jumonji domain-containing protein 3 (JMJD3). Both upregulation of *Jmjd3* gene and downregulation of PcG genes suppress H3K27me3 at the promoter of *p14* and *p16*, which triggers the activation of corresponding proteins and then exacerbates MSC aging [38]. On the other hand, HDAC and Twist1 exert their influence in MSC aging at the upstream of PcGs and JMJD3. Downregulation of *Twist1* gene expression in aged MSCs is followed by *Ezh2* suppression and E47 promotion. Then, the upregulated E47 protein directly binds to the *p16* promoter to increase its transcriptional activity, producing a synergistic action with insufficient EZH2 protein [39]. In comparison to *Twist1*, the regulatory mechanism of HDAC is more specific. In normal cells, HDAC restrains *Jmjd3* expression through deacetylating histone near its promoter region and oppositely promotes PcG proteins and c-MYC activity via the RB/E2F pathway. However, in aging MSCs, HDAC deficiency induces hypophosphorylation of retinoblastoma-like protein (RB). This prompts RB to bind to E2F and further compromises the overall expression of PcGs genes. Finally, alterations of JMJD3 and PcGs level bring about cell cycle arrest by demethylating H3K27 at the *p1*6^INK4A^ promoter [38]. Lee et al. also reported that HDAC inhibitors valproic acid and sodium butyrate both promote the acetylation of histone H3 and H4 to activate the transcription of *p2*1^CIP1/WAF1^, but the *p16* expression level remains unchanged unexpectedly [40]. By contrast, another group found that low concentrations of HDAC inhibitor largazole or trichostatin A induce improved proliferation, suppressed differentiation, and delayed aging of hUCSCs. The underlying mechanism is based on histone H3 acetylation and methylation around *Tert*, *Nanog*, *Oct4*, *Alp*, *Opn*, and *Cxcr4* genes [41]. SIRT6 protein is another regulator with HDAC activity. As an NAD-dependent H3K9 and H3K56-specific deacetylase, SIRT6 deficiency causes acetylated H3K56 accumulation and compromised recruitment of RNA polymerase II (RNAP II) complex to heme oxygenase 1 (*Hmox-1*) gene promoter. More importantly, without the cooperation of SIRT6, expression of *RNAP II*, *Nrf2*, and *Hmox-1* genes declines, resulting in impaired cellular redox homeostasis [42].

In terms of diseases, an imbalance between histone modifications of osteogenic and lipogenic genes is a possible mechanism. Intriguingly, as for histone modification, the regulatory effects of the same factor on osteogenic differentiation or adipogenic differentiation are not necessarily opposite. In other words, factors that promote adipogenesis may either inhibit or promote the biological osteogenic process [43]. For instance, mixed lineage leukemia protein (MLL), general control non-derepressible5 (GCNs, namely, KAT2A), and P300/CBP-associated factor (PCAF, namely, KAT2B) can promote both osteogenesis and lipogenesis, while HDAC1 can inhibit both. Moreover, SET domain bifurcated 1 protein, lysine-specific demethylase 4B/6B (KDM4B/6B), and HDAC3 all promote osteogenesis but inhibit lipogenesis; oppositely, EZH2 and HDAC6 promote lipogenesis but inhibit osteogenesis [43, 44]. Thus, homeostasis of bone tissues relies largely on coordinating and orderly expression in spatial-temporal dimensions. And histone modification dysregulation in osteoporosis is closely related to the break of balance among associated regulatory factors. For instance, in osteoporosis, the upregulated EZH2 and KDM5A and downregulated absent, small, or homeotic 1-like (ASH1L) genes suppress Wnt and Runx2 pathways by altering H3K4me3 and H3K27me3 levels [45–47]. Similarly, H3K9 acetyltransferase GCN5 and PCAF (namely, KAT2A and KAT2B) gliding deacetylates H3K9 on the promoter of *Wnt*, *BMP*, and *Runx2* genes [48–50]. As to the mechanism of oculofaciocardiodental (OFCD) syndrome characterized by extremely long dental roots and craniofacial defects, recruitment restriction of KDM mediated by BCL-6 corepressor increases the H3K4me3 level and promotes upregulation of AP-2*α*, whose osteogenesis-fortifying function is overactivated, leading to osteogenic hyperfunction in OFCD syndrome [51].

### 2.3. Chromatin Remodeling in MSC Aging and Osteoporosis

In a narrow sense, chromatin remodeling is an ATP-dependent process catalyzed by chromatin-remodeling complexes. The core component of the complexes is an ATPase subunit from the SNF2 family including SWI/SNF (switch/sucrose nonfermentable) and INO80 [SWI2/SNF2 related (SWR)] subfamilies [52]. In a broad sense, all factors that bring about chromatin structural alterations, including the relaxing or packing of chromatin by histone modification, contribute to chromatin remodeling (Table 3).

During the process of aging, the protein encoded by Brahma-related gene 1 (*Brg1*), the ATPase subunit of SWI/SNF chromatin remodeling complex, has been regarded as an essential factor in global modulation. Both the upregulation and downregulation lead to acceleration of cell senescence. On the one hand, when *Brg1* is silenced, the chromatin compaction cannot be completed. This facilitates DNMT recruitment and methylation at *Nanog* promoter and eventually induces transcription inhibition [53]. Besides, *Brg1* insufficiency-induced senescence is also linked to *γ*-isoforms of heterochromatin formation and p53 activation-induced cell cycle arrest [53, 54]. On the other hand, overexpression of *Brg1* also induces an increasing portion of programmed cell death, despite the fact that the specific mechanism is not clarified [55].

Apart from chromatin remodeling complexes, factors directly related to the chromatin structure, such as condensin and KRAB-associated protein 1 (KAP1), also contribute to chromatin remodeling. It has been reported that condensin I/II can alleviate DNA damage by chromatin reorganization in normal cells. However, hypermethylation around *NCAPD2/NCAPG2*, the encoding genes of the core components of condensin, leads to condensin shortage and DNA damage accumulation during aging [56]. Loss of heterochromatin is proved to be a potential cause of MSC aging [57]. For example, abnormality of the heterochromatin component KAP1 promotes MSC aging via a chromobox4- (CBX4-) dependent manner. When CBX4 declines in aged hMSCs, fibrillarin (FBL) and KAP1 cannot be recruited at nucleolar rDNA, leading to excessive expression of rRNAs, then trigger detrimental ribosome biogenesis and destabilize nucleolar heterochromatin [58].

As to osteoporosis, the INO80 chromatin remodeling complex interacts with WD repeat-containing protein 5 (Wdr5) protein that catalyzes H3K4me3 formation to positively regulate the canonical Wnt pathway. Correspondingly, after *INO80* gene is knocked down, the osteogenic potential of MSCs decreases both in *in vitro* and in ectopic transplantation models, reproducing a similar phenotype as osteoporosis [59].

In general, chromatin remodeling is intertwined with DNA methylation and histone modification; the DNA and histone modification status directly determines the accessibility and structure of chromatin. Histone acetylation may “open” chromatin by neutralizing the positive charges of lysine to increase site exposure of the surrounding negatively charged DNA [60]. Meanwhile, histone methylation modulates the synthesis of chromatin remodeling-related proteins [56]. Thus, the functional importance of the interactive mechanism is to realize orderly integration and feedback of all three processes.

### 2.4. mRNA Modification in MSC Aging and Osteoporosis

There are only a few studies about mRNA modification of MSCs during aging. Even so, the biological activity of MSCs in a normal bone is closely related to RNA N^6^-methyladenosine (m^6^A) modification. Osteogenesis induced by methyltransferase like 3 (METTL3) is counterbalanced with lipogenesis promoted by demethyltransferase fat mass and obesity-associated protein (FTO) [61]. During the aging process, expression of *Fto* gene increases and inhibits m^6^A formation on *Pparγ* mRNA, which results in lipogenesis through the GDF11-FTO-PPAR*γ* axis [62]. However, the adipogenesis and osteoporosis process could be prevented by METTL3 application [63].

### 2.5. ncRNA in MSC Aging and Osteoporosis

#### 2.5.1. ncRNA in MSC Aging

Alterations of miRNA and long noncoding RNA (lncRNA) abundance are closely associated with the aging process of MSCs either *in vivo* or *in vitro*. Researchers compared miRNA profiles in aged MSCs with younger generations to figure out up- or downregulated miRNA types (Table 4). Data from different organizations varied widely, probably because of different sources of MSCs or the experimental conditions [64–66]. Notably, the variation among MSC miRNA expression profiles from different tissues indicates that their regulatory mechanisms are relatively tissue-specific [67, 68].

Some upregulated miRNAs maintain senescent cells in a proliferation-disabled state by binding to the transcripts of genes related to the cell cycle. For instance, miR-22 and miR-485-5p directly target the cyclin-dependent kinase regulatory subunit 1 gene, thus impeding synthesis and function of CDK and cyclin B and cause G2/M phase arrest [69]. miR-34a also targets *CDK2*, *CDK4*, *CDK6*, *cyclin D*, *cyclin E*, and *RBP2* to hinder self-renewal ability [70]. In addition, miR-31a-5p can bind to the 3′UTR of *E2F2* mRNA and bring about senescence-associated heterochromatin foci formation in aged rat BMSCs [71]. Besides, *CNOT6* encodes deadenylase subunits of the Ccr4-Not complex; miR-29c-3p-induced CNOT6 downregulation can induce responsive elevation of *p53*, *p21*, and *p16* expression followed by arrest of cell cycle [72, 73].

Correspondingly, with replicative pressure due to serial passages, miRNAs that play roles in repressing senescence-inducing proteins are downregulated. Downregulation of these miRNAs initially leads to dysregulation of global gene regulatory network and eventually fosters the aging process. For example, downregulation of miR-10a makes it insufficient to suppress senescence-inducing function of Krüppel-like factor 4 (KLF4) [74, 75]. And intriguingly, downregulation of miR-17 family (including miR-17, miR-20b-5p, and miR-106a-5p) regulates the activities of various genes, including Smad ubiquitination regulatory factor-1 (*Smurf1*), *p21*, *CCND1*, and *E2F1* genes in aging [76, 77]. Besides, other downregulated miRNAs including miR-543, miR-590-3p, and miR-24a have been discovered to modulate p18/p21 and p16 activity separately [78, 79]. In addition, downregulation of miR-199b-5p also promotes cell cycle arrest indirectly via laminin gamma 1 [80].

Accumulation of reactive oxygen species (ROS) is another important mechanism of stem cell aging. Several miRNAs influence the production or elimination of ROS epigenetically. In younger cells, the amount of peroxisome is restricted by pexophagy. However, in aged cells, upregulated miR-142 targets the endothelial PAS domain protein (*Epas1*) gene, a positive regulator of pexophagy, inducing ROS accumulation [81]. Moreover, miR-155-5p can repress antioxidant genes by targeting the common transcription factor CCAAT/enhancer-binding protein *β* (*C/EBP-β*) gene and then induces ROS generation [82]. miR-182 antagonizes osteoblast proliferation and differentiation by targeting *FoxO1* gene, which protects hBMSCs from ROS-induced harm [83]. Besides, miR-183-5p, which belongs to the same cluster to miR-182, also increases in extracellular vesicles (EVs) derived from the bone marrow of aged mice. It accelerates cell senescence by impairing HMOX-1 protein's responsive capacity to oxidative stress [84].

miRNA also affects cell aging through mitochondrial or telomere mechanisms. miR-34a targeting *Sirt1* and miR-155 targeting calcium-binding protein 39 (*Cab39*) are all contributors in mitochondrial mechanisms [85–90]. When it comes to the telomere hypothesis of aging, miR-195 binds to the 3′UTR of *Tert* mRNA, thus preventing TERT protein from repairing the shortened telomeres due to replicative senescence [91].

miRNAs such as miR-27, mi-R141-3p, and miR-1292 can also induce aging through multiple cellular pathways involved in cell differentiation and metabolism. The more detailed information is listed in Table 4 [92–100].

As for lncRNAs, although they are regarded as crucial modulators of MSC-mediated ectopic tissue regeneration, only a few studies have reported the influence of lncRNAs on aging. lncRNAs regulate gene expression in diverse manners, such as serving as scaffolds to facilitate the assembly of specific transcriptional complexes or acting as sponges to reduce the availability of targeted miRNAs [101]. For instance, upregulated lncRNA-HOTAIR has been found to bring about senescence-associated changes, including differential expression of specific genes and abnormal DNA methylation by facilitating triple-helix DNA-DNA-RNA formation [102]. Besides, lncRNA-Bmncr serves as a scaffold to facilitate the interaction of Abelson murine leukemia viral oncogene homolog 1 protein and transcriptional coactivator with PDZ-binding motif (TAZ), guaranteeing the assembly of the TAZ and *Runx2*/*Pparγ* transcriptional complex to inhibit MSC adipogenic differentiation. When lncRNA-Bmncr expression decreases in aging MSCs, the tendency of lipogenesis increases [103].

#### 2.5.2. ncRNAs in Osteoporosis

It has been widely reported that miRNAs and lncRNAs play significant roles in maintaining the balance between osteogenesis and adipogenesis. Scholars have summarized that miRNAs were involved in osteogenic regulation mainly through two patterns: affecting *Runx2* expression via canonical Wnt pathway, TGF-*β* pathway, and BMP pathway or directly targeting *Runx2* or *Osx* genes. On the other hand, ncRNAs influence adipogenesis through PPAR*γ* and C/EBP-*α* [104, 105]. When these miRNA expression levels change and differentiation balance is broken, bone diseases such as osteoporosis will occur. For instance, it has been reported that in osteoporosis, upregulated miR-23b, miR-3077-5p, miR-212, and miR-384 inhibit osteogenesis by directly targeting *Runx2* gene [106–108], while in ONFH, upregulated miR-596 and miR-708 hinder osteogenesis by binding to *Smad3* transcripts to suppress *Runx2* gene expression [109, 110]. In addition, miRNAs such as miR-542-3p, miR-21, and miR-27 target negative regulators in osteogenesis-related pathways; thus, their downregulation also leads to MSC lineage commitment transition from osteocytes to adipocytes [111–113]. In addition to modulating MSC differentiation, miR-181a and miR-1263 also influence skeletal homeostasis by regulating cell apoptosis through FasL accumulation and Mob-1/YAP/Hippo, respectively, [114, 115]. Besides, downregulated miR-21a is insufficient to inhibit phosphatase and tension homolog (*Pten*) gene expression, and abundant PTEN protein downregulates the AKT pathway to induce osteocyte apoptosis in glucocorticoid-induced osteonecrosis [116].

lncRNAs often exert their pathogenic effect via miRNAs or other ncRNAs. For example, miR-143, a direct inhibitor of *Osx* gene, is suppressed by lncRNA-MALAT1 in normal MSCs. However, in osteoporosis patients, downregulation of lncRNA-MALAT1 leads to decreased *Osx* gene expression and loss of bone mass [117]. Another team also proved that lncRNA-MALAT1 acts as a sponge of miR-34c to increase the expression of special AT-rich sequence binding protein 2 (SATB2), which is conducive to restoring osteogenesis in osteoporosis conditions [118]. Besides, elevated lncRNA-ORLNC1 endogenously competes with miR-296 and eliminates miR-296's suppression of *Pten* gene, which is a negative regulator of osteogenesis [119]. Moreover, in ONFH, upregulated lncRNA-DEPTOR binds to lncRNA-MEG3 promoter and prevents it from activating BMP4 pathway [120]. It is worth mentioning that in the process of skeletal diseases, alteration of lncRNA expression may work against bone destruction. For example, lncRNA-H19 inhibits MSC proliferation and osteogenic differentiation via suppressing miR-196-3p when estrogen exists. However, in postmenopausal osteoporosis, *H19* expression level decreases [121]. Similarly, in ONFH, lncRNA-HOTAIR is upregulated and promotes osteogenesis via the miR-17-5p/Smad7 pathway [122].

In general, existing research reveals that in terms of mechanism, ncRNAs primarily regulate cell cycle or affect aging-related factors including ROS, telomere, and mitochondria to induce MSC aging. In the process of skeletal diseases, epigenetic factors promote disease progression through biased differentiation and cell apoptosis. Specifically speaking, the dysregulation of miRNA and lncRNA forecasts the dysfunction of MSCs, and notably, modulative effects of ncRNAs are not directly realized in a unidirectional manner.

### 2.6. The Interplay of Different Epigenetic Factors in MSC Aging

As we mentioned in the previous parts, MSC aging is epigenetically marked by heterochromatin loss, altered DNA methylation profile, and organized histone modification. Actually, it should be particularly noted that epigenetic factors do not perform their functions in a parallel and independent fashion. It has been extensively reported that a multitude of them mutually intertwine and influence. Firstly, DNA methylation and histone modification are closely related in many aspects. In aged MSCs, hypomethylated CpG sites always come together with H3K4me, and both of them are signs of increased transcription activity, while DNA regions enriched in hypermethylated CpG sites mainly overlap with the repressive chromatin marks H3K27me3 and H3K9me3, synergistically inhibiting transcription activity [22, 25]. Moreover, DNA demethylation-induced PcG downregulation can regulate H3K27 methylation [28]. Secondly, histone modifications can alter chromatin structure not only by influencing histone-DNA, histone-histone interactions, and chaperone-histone binding [60] but also through modulating the synthesis of chromatin remodeling-related proteins such as condensin [56]. Last, DNA methylation also influences chromatin structure by regulating transcription factors, such as *SMARCs* and *SIN3A* [24]. Besides, it has been discovered that considerable ncRNAs interplay with complicated molecular networks and pretranscriptional epigenetic marks in the aging process. For instance, depletion of DNA demethylase TET causes accumulated miR-297; miR-188 inhibits HDAC9-mediated histone deacetylation; and miR-31a-5p can bring about senescence-associated heterochromatin foci formation in aged rat BMSCs [34, 71, 95]. Pursuit in epigenetics about MSCs has never slowed down. It is believable and desirable that more interactive relationships between different epigenetic marks could be established and elaborated. However, according to existing researches and experimental technology, it is difficult and one-sided for us to rank a certain epigenetic mark as a more critical inducing factor for MSC aging. Instead, it is most likely that they function as an interlaced system in coordination and order. Although it has been reported that intervention of a single factor of epigenetic marks could delay or reverse MSC aging to some extent, we believe that other types of accompanied epigenetic regulation may initiate in the biological effect, and even imaginably, proper combinational modulation of two or more epigenetic marks could upgrade the efficacy of therapy for MSC-related diseases.

## 3. Application of Epigenetic Regulation in Skeletal Diseases and Engineering Regeneration

**Figure 2:**
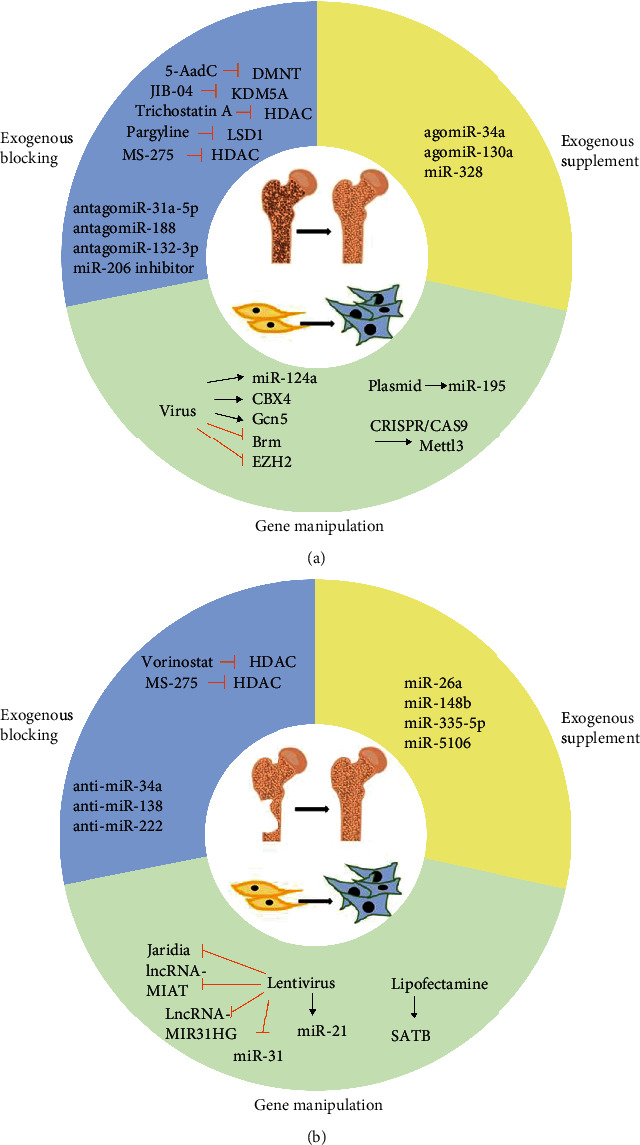
Application of epigenetic regulation in (a) skeletal diseases and (b) bone regeneration. Exogenous blocking or supplement and direct gene manipulation are separately used in both conditions.

MSCs are an essential source for cell-based bone regeneration. The premise of bone regeneration is to maintain MSC stemness and promote their osteogenic differentiation. As mentioned above, it has been demonstrated that epigenetic markers and modifiers play fundamental roles in aging and diseases through modulating MSC function. Thus, MSC-mediated therapeutic or regenerative strategies based on epigenetic principle possess enormous potential in the treatment of aging-related bone disorders and defects. Despite the lack of experiments that mediate bone regeneration by means of epigenetic regulation in aging models, great efforts have been made in corresponding explorations with normal animal models. In turn, tentative application of epigenetic therapies *in vivo* further reinforces our understanding of the intrinsic mechanisms and makes it possible to realize our clinic utilization in the future. So far, there are three kinds of commonly used epigenetic interventions of tissue diseases or regeneration *in vivo*: exogenous inhibition of negative epigenetic regulators, exogenous supplement of positive regulators, and direct gene manipulation (Figure 2).

### 3.1. Exogenous Blocking

One common method is to block epigenetic factor-induced MSC aging and diseases by using exogenous inhibitors. For bone tissue regeneration in nonaging animal models, a combination of collagen sponge and HDAC1/4 inhibitor MS-275 exerts a promotive effect in rat critical-sized calvarial defect healing [123]. Moreover, intraperitoneal injection of MS-275 avoids delayed cranial suture closure in Runx2-null mice [124]. Similarly, when vorinostat, another HDAC1 inhibitor, was intraperitoneally injected into mice, the number of osteoblasts in endocortical bone increased and OCN level in serum rose [125]. Except for that, miRNA-mediated bone regeneration usually proceeds with specific biomaterials [126]. For example, when hMSCs transfected with anti-miR-34a, anti-miR-138, or anti-miR-222 by lipofectamine are loaded on a hydroxyapatite/tricalcium phosphate ceramic powder, the ectopic bone formation on complex scaffolds increases compared to the untransfected group [127, 128]. Moreover, anti-miR-222 also manifests a promotive effect toward bone defect healing when directly loaded on the atelocollagen scaffold [129].

Simultaneously, great progress has been made in MSC-dependent epigenetic therapeutics aimed at aging-related skeletal diseases. For histone acetylation, MS-275 subcutaneous injection rescues NF-*κ*B-induced rapid bone loss by interrupting interactions between HDACs and DExH-box helicase Dhx36, which inhibits tissue-nonspecific alkaline phosphatase (TNAP) activity [123]. Pretreatment with KDM5A inhibitor JIB-04 partially rescues bone loss during osteoporosis by increasing the H3K4me3 level on the *Runx2* promoter [45]. And HDAC inhibitor trichostatin prospers osteogenesis of rat adipose tissue-derived stem cells (hADSCs) by histone modifying on *Runx2* promoter [130]; LSD1 inhibitor pargyline rescues osteogenic ability of BMSCs under osteoporosis conditions by modulating H3K4 methylation at the promoter region of *Ocn* and *Runx2* genes [131]. Notably, miRNA inhibitors are also applied in osteoporosis treatment. For example, injection of antagomiR-31a-5p or antagomiR-188 into bone marrow cavity significantly alleviates fat accumulation and remedies bone loss in aged mice [71, 95]. Moreover, atagomiR-132-3p delivered by a bone-targeted (AspSerSer)_6_-cationic liposome system silences miRNA-132-3p expression in bone tissues, thus effectively preserving bone mass, bone structure, and strength in hindlimb-unloaded mice [132].

### 3.2. Exogenous Supplement

ncRNAs or their mimics can be exogenously supplemented to delay or reverse disease progression. It has been extensively reported that loading MSCs transfected by proosteogenic miRNAs (such as miR-26a, miR-148b, miR-5106, miR-335-5p, or their mimics) on biomaterials is an effective strategy to promote bone regeneration [133–136].

With regard to skeletal disease treatment, agomiR-130a intravenously injection reduces bone loss in elderly mice by targeting *Smurf2* and *Pparγ* genes [137, 138], while collagen-based hydrogel containing agomir-34a elevates bone volume in mouse radiational bone injury area by downregulating *Notch1* expression in BMSCs [139]. In addition, miR-328 is the antagonist of *Axin1* gene, whose product AXIN1 protein is an inhibitor of Wnt signaling pathway. Thus, application of apoptotic bodies containing miR-328 significantly ameliorates osteopenia in OVX mice [140].

### 3.3. Gene Manipulation

In addition to the above two methods, virus transfection and CRISPR/Cas9 are applied to realize direct gene manipulation. Jumonji AT-rich interactive domain 1A (JARID1A) protein is a KDM5A component participating in *Runx2*-related H3K4 demethylation. Compared with the control group, scaffolds containing BMSCs transduced with si-Jarid1a increase bone volume and mineral density during the process of calvarial defect healing [141]. Besides, SATB2 protein is a nuclear matrix protein involved in chromatin remodeling, and the *Satb2* gene overexpression by lipofectamine transfection enhances skeletal tissue regeneration and mineralization in mouse mandibular bone defects [142]. Moreover, anti-miR-31-expressing BMSCs/poly (glycerol sebacate) complex and miRNA-21-modified BMSCs/*β*-tricalcium phosphate composite both bring higher bone regeneration rate in rat bone defects [143, 144]. Similarly, knockdown lncRNA MIR31HG or MIAT with lentivirus significantly enhances ADSCs' bone formation capacity when implanted subcutaneously with biomaterials [145, 146].

As to therapeutic application in diseases, injection of lentiviruses encoding CBX4 protein into the joint capsules leads to upregulation of proliferation, bone growth-associated genes, and downregulation of inflammation and cell death-related genes [58]. Similarly, mammalian brahma (BRM) protein is a component of SWI/SNF complex with ATPase activity. Knockdown of *Brm* gene in mice helps it resist aging-related osteoporosis and reduces adiposity in bone marrow [147]. Lentivirus is also used to alter histone acetylation and methylation level in osteoporosis. In OVX mice, injection of lentiviruses expressing *Gcn5* gene restores endogenous BMSC osteogenic potential by increasing H3K9ac on the promoters of Wnt genes [48]. Except for that, knockdown of *Ezh2* gene by lentivirus-expressing shRNA decreases H3K27me3 on *Wnt* genes, reversing the abnormal MSC adipogenic lineage commitment in osteoporosis [47]. Moreover, when RNA N6-methyltransferase Mettl3 gene is knocked in transplanted MSCs with CRISPR/Cas9 and Cre/LoxP, mice are protected from OVX-induced osteoporosis [63]. When it comes to ncRNAs, bone defects completely healed with transplantation of BMSCs expressing miR-214 sponges transduced by baculovirus [148].

## 4. Conclusion

Epigenetic regulation of MSCs occurs in several steps of transcription, including chromatin remodeling, DNA methylation, and histone modification, and posttranscription, including mRNA processing and ncRNA regulation. Epigenetic markers and modifiers have been proved to play indispensable roles in MSC aging and fundamental homeostasis *in vivo*, both of which are related to the pathogenesis of tissue disorders in aging and diseases. Initial experimental attempts, roughly according to epigenetic clues, have been carried out to delay MSC aging or rejuvenate senescent MSCs, which is aimed at enhancing their self-renewal capacity and correct biased differentiation lineage. However, there remain several obstacles for translational application, including lack of sequential identification of spatiotemporal epigenetic alteration, and difficulties in precise translational intervention *in vivo*. Hopefully, many revolutionary technological progresses emerged just in the past years, including single-cell epigenomic analysis and CRISPR/Cas9, cell transplantation, and regenerative biomaterials. In this context, therapeutic or regenerative strategies based on epigenetic regulation of MSC aging stand a tremendous chance to restore MSC homeostasis *in vivo* and even boost translational application in tissue regeneration, especially among the elderly or people with bone diseases.

## Figures and Tables

**Table 1:** DNA methylation in MSC aging and related diseases.

Regulatory factors	Mechanism	*In vivo* or *in vitro*	Consequence	Materials	Ref.
*Senescence and aging*					
DNMT1↓ DNMT3b↓	Decreases methylation in the promoter region of miRNA targeting *Ezh2* to decrease *Ezh2* expression, thus inhibiting repressive H3K27me3 formation at *p16*'s promoter; directly decreases methylation in CDK inhibitor genes *p16* and *p21*	*In vitro*	Aging	hUCSCs	[28]
DNMT↓	Insufficient to methylate *Tert* promoter; thus upregulated TERT would repair the shortened telomeres with replication	*In vitro*	Antisenescence	hBMSCs	[30]
*Skeletal diseases*					
DNMT1 changes	Aberrant methylation of *ABCB1* gene leads to a dysregulation of glucocorticoid	*In vitro*	ONFH	hBMSCs	[33]
TET1 and TET2↓	The depletion of TET causes impeded demethylation of *P2rX7* promoter and incapable exosome release, which leads to intracellular accumulated miR-297 targeting Runx2 signaling pathway	Both	Osteoporosis	hBMSCs, mouse BMSCs, mouse model	[34]

**Table 2:** Histone modification in MSC aging and related diseases.

Epigenetic changes	Factors	Mechanism	*In vivo or in vitro*	Consequence	Material	Ref.
*Senescence and aging*						
Histone acetylation	HDAC↓	Directly upregulates JMJD3 and indirectly downregulates *PcGs* through RB/E2F pathway to inhibit H3K27me3 at *p16^INK4A^*	*In vitro*	Aging	hADSCs, hUCSCs	[38]
Histone acetylation	HDAC↓	Promotes the transcription of *p21^CIP1/WAF1^* through increasing H3 and H4 acetylation	*In vitro*	Aging: decreased differentiation ability and proliferation rate	hADSCs, hUCSCs	[40]
Histone acetylation	SIRT6↓	Insufficient SIRT6 causes increased H3K56ac and compromised recruitment of RNAP II complex to *Hmox-1* gene promoter, leading to decrease in *Hmox-1* expression and impaired cellular redox homeostasis	Both	Senescence, dysregulated redox metabolism, and increased sensitivity to oxidative stress	Human embryoid bodies MSC, mouse model	[42]
Histone methylation	TWIST1↓	Insufficient to prevent senescence by recruiting EZH2 and form repressive H3K27me3 at *p16/p14* promoters; upregulates *E47* that binds to *p16* promoter and promotes transcription activity	*In vitro*	Senescence	hBMSCs	[39]
Histone methylation	BMI1↓	Fails to recruit and stabilize PRC2 which protects H3K27me3 of *p16^INK4A^*	*In vitro*	Aging	hADSCs, hUSCSs	[38]
Histone methylation	EZH2↓	Fails to methylate H3K27 as catalytic subunit of PRC2; insufficient H3K27me3 cannot suppress *p16* and *p14* expression	*In vitro*	Aging	hADSCs, hUSCSs	[38]
Histone methylation	G9a↓	(Unclear)	*In vitro*	Aging: decreased differentiation ability and proliferation rate	Rat BMSCs	[149]
*Skeletal diseases*						
Histone methylation	EZH2↑	Promotes H3K27me3 on *Wnt1*, *Wnt6*, and *Wnt10a* promoters to silence Wnt signaling pathway	Both	Osteoporosis	hBMSCs, mouse BMSCs, mouse model	[47]
Histone methylation	KDM5A↑	Increases H3K4me3 levels on promoters of *Runx2*	Both	Osteoporosis	hBMSCs, mouse BMSCs, mouse model	[45]
Histone methylation	ASH1L↓	Fails to mediate H3K4me3 recruitment at the transcription start sites of *Osx*, *Runx2*, *Sox9*, and *Creb* genes	Both	Osteoporosis	hBMSCs, mouse BMSCs, mouse model	[46]
Histone methylation	KDM2B↓	Unable to be recruited to the promoter of *AP-2α* and inhibit AP-2*α* expression via removing H3K4me3	Both	Oculofaciocardiodental (OFCD) syndrome	hBMSCs, mouse BMSCs, mouse model	[51]
Histone acetylation	GCN5 (KAT2A)↓	Insufficient to increase H3K9 acetylation on the promoters of *Wnt* genes	Both	Osteoporosis	hBMSCs, mouse BMSCs, mouse model	[48]
Histone acetylation	PCAF (KAT2B)↓	Insufficient to acetylate H3K9 at promoters of *BMP2*, *BMP4*, *BMPR2B*, and *Runx2*	Both	Osteoporosis	hBMSCs, mouse BMSCs, mouse model	[49]

**Table 3:** Chromatin remodeling in MSC aging and related diseases.

Regulatory factors	Mechanism	*In vivo or in vitro*	Material	Consequence	Ref.
*Senescence and aging*					
BRG1↓	Inhibits *Nanog* gene expression by facilitating DNMT recruitment and methylation; induces *γ*-form heterochromatin formation and p53 pathway activation	*In vitro*	hBMSCs	Senescence	[53, 54]
Condensin↓	Fails to alleviate DNA damage by chromatin reorganization	Both	hBMSCs, mouse BMSCs, mouse model	Aging, bone aging	[56]
KAP1↓	Cannot be recruited by insufficient CBX4 at nucleolar rDNA, enhancing the excessive expression of rRNAs and destabilizing nucleolar heterochromatin	Both	hMSCs derived from embryonic cell culture, mouse BMSCs, mouse model	Premature cellular senescence, osteoarthritis	[58]
*Skeletal diseases*					
INO80↓	Incapable of interacting with Wdr5 that catalyzes H3K4me3 formation, which promotes Wnt pathway activity	Both	hBMSCs, mouse model	Osteoporotic phenotypes	[59]

**Table 4:** ncRNAs in MSC aging and related diseases.

Regulatory factors	Mechanism	*In vivo* or *in vitro*	Consequence	Material	Ref.
*Senescence and aging*					
miR-10a↓	Insufficient to target *Klf4* and repress its function	Both	Senescence, decreased differentiation	hBMSCs	[74, 75]
miR-20b-5p and miR-106a-5p↓	Insufficient to inhibit Smads/p21/CDK/E2F pathway, which alleviates suspension of DNA synthesis during oxidative stress-induced premature senescence	*In vitro*	Premature senescence	hBMSCs	[77]
miR-22 and miR-485-5p↑	Targets *CKS1* to downregulate CDK1 and cyclin B	*In vitro*	Senescence	SHED	[69]
miR-31a-5p↑	Targets *E2F2* and promotes SAHF formation	Both	Senescence	Rat BMSCs	[71]
miR-27b↑	Upregulates *p16* expression and MAPK pathway activation	*In vitro*	Senescence	Pig ADSCs	[98]
miR-29c-3p↑	Targets *CNOT6* thus inducing senescence via p53/p21 and p16/pRB pathways	*In vitro*	Senescence	hBMSCs	[72, 73]
miR-34a↑	Reduces *CDK2*, *CDK4*, *CDK6*, and *cyclin D* and *E* expression to hinder the SOX2-related self-renewal ability	*In vitro*	Senescence	hADSCs	[70]
miR-34a↑	Targets *Sirt1* to induce senescence via Sirt1/FoxO3a pathway, induces mitochondrial dysfunction	*In vitro*	Senescence and intrinsic apoptosis	Mouse BMSCs, rat BMSCs	[89, 90]
miR-141-3p↑	Targets *Zmpste24* transcripts, causing prelamin A accumulation in nuclear envelope and intracellular DNA damage	*In vitro*	Senescence	hUCSCs	[92]
miR-141-3p↑	Targets *YAP* to inhibit proliferation and accelerate senescence	*In vitro*	Senescence	Human papilla apical stem cells	[93]
miR-142↑	Targets *Epas1* to downregulate pexophagic activity and induce ROS accumulation	*In vitro*	Aging	Mouse BMSCs	[81]
miR-155-5p↑	Targets *Cab39* and then reduces mitochondrial fission and increases mitochondrial fusion via the Cab39/AMPK signaling pathway	Both	Aging	Mouse model, hBMSCs	[86]
miR-155-5p↑	Targets *Bag5* that encodes partner protein of PINK1, to inhibit mitophagy and dysfunctional mitochondria elimination	*In vitro*	Aging	hBMSCs	[87]
miR-155-5p↑	Targets the common transcription factor *C/EBP-β* thus repressing antioxidant genes and inducing ROS production	Both	Aging	hBMSCs/mouse BMSCs	[82]
miR-182↑	Targets *FoxO1*, which is critical to protecting cells from ROS	*In vitro*	Aging, decreased proliferation, and osteogenesis	hBMSCs	[83]
miR-183-5p↑	Targets *Hmox-1* to impair response to oxidative stress	*In vitro*	Senescence	Mouse BMSCs	[84]
miR-188↑	Targets *HDAC9* and *RICTOR*	Both	Aging, decreased proliferation	Mouse BMSCs, mouse model	[94, 95]
miR-195↑	Targets *Tert* and prevents TERT to repair the shortened telomeres with replication	*In vitro*	Aging	Mouse BMSCs	[91]
miR-199b-5p↓	Insufficient to repress LAMC1	*In vitro*	Senescence	hBMSCs	[80]
miR-206↑	Targets *Alpl*, which is essential for the intracellular ATP level and AMPK pathway	Both	Premature senescence	Rat BMSCs, rat model	[96]
miR-363-3p↑	Targets *TRAF3*, which inhibits adipogenic differentiation and senescence	*In vitro*	Senescence, upregulated adipogenesis	Rat BMSCs	[99]
miR-486-5p↑	Targets *Sirt1*	*In vitro*	Senescence	hADSCs	[85]
miR-543 and miR-590-3p↓	Insufficient to target *AIMP3/p18* to inhibit expression, inducing an increase in CDK inhibitors p16^INK4A^ and p21^CIP1/WAF1^	*In vitro*	Senescence	hUCSCs	[78]
miR-1292↑	Targets *Wnt* receptor FZD4, thus hindering the Wnt/*β*-catenin/TCF/LEF1 pathway	*In vitro*	Senescence, downregulated osteogenesis	hADSCs	[100]
lncRNA-Bmncr↓	Insufficient to serve as a scaffold to facilitate the interaction of ABL and transcriptional coactivator with TAZ, hindering the assembly of the TAZ and RUNX2/PPAR*γ* transcriptional complex	Both	Aging, transition from osteogenesis to adipogenesis	hBMSCs, mouse BMSCs	[103]
lncRNA-HOTAIR↑	Modulates senescence-associated changes in gene expression and DNA methylation via triple helix DNA-DNA-RNA formation	*In vitro*	Senescence	hBMSCs	[102]
*Skeletal diseases*					
miR-21↓	Insufficient to target *Spry1*, which negatively regulates osteogenesis via FGF and MAPK	Both	Osteoporosis	hBMSCs, mouse model	[112]
miR-21a↓	Insufficient to target *Pten* and PTEN downregulates AKT pathway to induce osteocyte apoptosis	Both	Glucocorticoid-induced osteonecrosis	hUCSCs, mouse model	[116]
miR-23b↑	Targets *Runx2*	Both	Osteoporosis	hBMSCs, mouse model	[106]
miR-27↓	Insufficient to target *Mef2c*, which facilitates the adipogenic differentiation	Both	Osteoporosis	hBMSCs, mouse model	[113]
miR-181a↓	Leads to the accumulation of FasL from BMSCs, followed by CD4+ T cell apoptosis	Both	Osteoporosis	Mouse BMSCs, mouse model	[115]
miR-212 and miR-384↑	Targets *Runx2*	Both	Osteoporosis	Mouse BMSCs, mouse model	[108]
miR-542-3p↓	Insufficient to inhibit *sFRP1* expression, which is a negative regulator of Wnt pathway	Both	Osteoporosis	HEK293T cells, rat BMSCs, rat model	[111]
miR-596↑	Targets *Smad3* to inhibit *Runx2* expression and osteogenesis	*In vitro*	ONFH	hBMSCs	[109]
miR-705 and miR-3077-5p↑	Respectively, targets *HOXA10* and *Runx2* mRNA, leading to MSC lineage commitment transition to adipocytes	*In vitro*	Osteoporosis	hBMSCs	[107]
miR-708↑	Targets *Smad3* to inhibit *Runx2* expression	*In vitro*	ONFH	hBMSCs	[110]
miR-1263↓	Insufficient to suppress Mob-1/YAP/Hippo signaling pathway-induced apoptosis	Both	Disused osteoporosis	hUCSCs, rat model	[114]
lncRNA-MALAT1↓	Insufficient to inhibit miR-143, whose target is *Osx*	*In vitro*	Osteoporosis	hBMSCs	[117]
lncRNA-ORLNC1↑	Endogenously competes with miR-296 and eliminates miR-296's suppression of *Pten*, which is a negative regulator of osteogenesis	Both	Osteoporosis	hBMSCs, mouse model	[119]
lncRNA-DEPTOR↑	Binds to ncRNA-MEG3's promoter and reduces its function to activate BMP4 pathway	Both	Osteoporosis	Mouse BMSCs, mouse model	[120]
lncRNA-H19↓	Insufficient to inhibit MSC proliferation and osteogenic differentiation via suppressing miR-19b-3p	*In vitro*	Inhibits osteoporosis	hBMSCs	[121]
lncRNA-HOTAIR↑	Suppresses miR-17-5p to elevate Smad7 pathway	*In vitro*	Inhibits ONFH	hBMSCs	[122]

ABL1: oncogene homolog 1; AIMP3: aminoacyl-tRNA synthetase-interacting multifunctional protein-3; CKS1: cyclin-dependent kinase regulatory subunit 1; Mef2c: myocyte enhancer factor 2c; LAMC1: laminin gamma 1; PRC2: polycomb repressive complex 2; SAHF: senescence-associated heterochromatin foci; sFRP1: secreted Frizzled-related protein-1; Spry1: sprouty homolog 1.
